# Establishing a novel sepsis-associated brain dysfunction model in zebrafish larvae

**DOI:** 10.3389/fimmu.2026.1862109

**Published:** 2026-10-29

**Authors:** Siqi Wu, Tao Zhang, Shengya Guo, Zhongyan Li, Xujie Pan, Yanfeng Huang, Lingling He, Yong Zhang, Ming Jiang

**Affiliations:** 1 Center for Genetic Medicine and Department of Otolaryngology-Head and Neck Surgery, The Fourth Affiliated Hospital, Zhejiang University School of Medicine, Hangzhou, Zhejiang, China; 2 Hunter BioInsight Technology, Inc., Hangzhou, China; 3 Hunter Biotechnology, Inc., Hangzhou, China; 4 State Key Laboratory of Natural Medicines, Department of Pharmacology, School of Pharmacy, China Pharmaceutical University, Nanjing, China

**Keywords:** disease model, ferroptosis, inflammation, larval zebrafish, sepsis-associated brain dysfunction

## Abstract

**Objective:**

As a common complication of sepsis, sepsis-associated encephalopathy (SAE) was observed with high mortality, but there were less effective therapeutic strategies due to disease complexity. This study aimed to establish and characterize a larval zebrafish model of sepsis-associated brain dysfunction (SBD) suitable for phenotypic assessment and compound screening.

**Methods:**

The SBD zebrafish larvae model was established by lipopolysaccharide (LPS) injection into the peritoneal cavity. Sepsis-induced inflammation of the intestine and brain was assessed using transgenic fluorescent larvae. The infection’s influence on intestine structure and brain function was monitored by HE staining, fluorescence staining, and behavioral assessment. Simultaneously, 16S rRNA sequencing and whole-larva transcriptomic profiling were conducted to characterize larva-associated microbial changes and systemic molecular responses. The expression of genes involved in inflammation, barrier function, ferroptosis as well as apoptosis was quantified via qPCR.

**Results:**

After LPS treatment for 24 h, there were significant neutrophil infiltration, increasing peristalsis, structural damage, and upregulated inflammatory gene expression in the intestine. Traced by using 10 kDa dextran-tetramethylrhodamine, the zebrafish larvae were observed with obviously increased fluorescence in the brain. In combination with downregulated tight junction genes, it suggested the breakdown of blood–brain barrier permeability. Simultaneously, we found increasing apoptotic cells, infiltrated neutrophils as well as imbalanced neurotransmitters in the larvae brain, and they exhibited reduced locomotor activity and altered color-preference/exploratory behavior. The 16S rRNA sequencing and transcriptome results showed restructuring of the larva-associated microbial community, including an increased relative abundance of *Aeromonas*. Whole-larva transcriptomic profiling identified a prominent ferroptosis-related signature, which was supported by increased ROS levels, altered ferroptosis-related gene expression, and mitochondrial changes. Increased lipid peroxidation provided additional support for ferroptosis-related alterations.

**Conclusion:**

We successfully developed a novel sepsis-associated brain dysfunction zebrafish larvae model. Based on its advantages of high throughput, rapidity, visualization, and facile gene edition, this model is expected to be beneficial for SAE drug discovery and disease mechanism exploration.

## 1 Introduction

Sepsis is a life-threatening syndrome caused by a dysregulated host response to infection and can ultimately lead to multi-organ dysfunction (1). More than 50% of patients with sepsis develop brain dysfunction, which may manifest as delirium, sickness behavior, memory disturbances, and cerebral ischemic changes. The presence of sepsis-associated encephalopathy (SAE) is associated with an increased risk of mortality (2, 3). Although advances in critical care have improved survival, neurological sequelae and cognitive impairment remain common among survivors (4). Therefore, effective approaches for the prevention and treatment of SAE are urgently needed.

SAE is defined as diffuse brain dysfunction occurring during sepsis in the absence of direct central nervous system infection (5). During sepsis, the mechanisms by which peripheral infection and systemic immune responses affect the central nervous system remain poorly understood. Neuroinflammation, blood–brain barrier (BBB) dysfunction, oxidative stress, and microglial activation have been implicated in the pathophysiology of SAE (6, 7). Recent studies have shown that SAE is associated with gut microbial dysbiosis (8, 9). The gastrointestinal system plays an important role in the pathophysiology of sepsis. Bidirectional communication between the intestine and the central nervous system occurs through the gut–brain axis. Sepsis-associated alterations in microbial composition and metabolism, together with intestinal barrier dysfunction, may influence neural, endocrine, immune, and metabolic signaling, potentially contributing to neuroinflammation and neurological dysfunction (10–12). Accordingly, modulation of the gut microbiota and maintenance of gut–brain axis homeostasis have been proposed as potential approaches for SAE management.

Cecal ligation and puncture (CLP) and intraperitoneal LPS administration are commonly used to establish rodent models of sepsis (13–15). However, their relatively high cost, considerable workload, and limited throughput restrict their application in large-scale drug screening. Larval zebrafish have attracted increasing attention because of their short developmental cycle, low cost, optical transparency, suitability for higher-throughput analysis, and ease of genetic manipulation. Zebrafish also possess conserved innate immune and inflammatory pathways and have been increasingly used to investigate infection- and inflammation-related diseases (16–19). *Mark Widder* and Anju M Philip previously developed zebrafish models of sepsis for drug screening (20, 21). In these studies, LPS was added to the embryo medium, and the experimental endpoints focused primarily on LPS-induced mortality rather than brain-related phenotypes. In the present study, we established a larval zebrafish model of sepsis-associated brain dysfunction (SBD) by microinjecting a defined dose of LPS into the peritoneal cavity (**Figure 1**). The term SBD was used because the measured endpoints, including BBB permeability, neuroinflammation, neurochemical alterations, and behavioral changes, reflect brain dysfunction in the larvae. This model may provide a practical platform for phenotypic drug screening and further investigation of sepsis-associated brain dysfunction.

**Figure 1:**
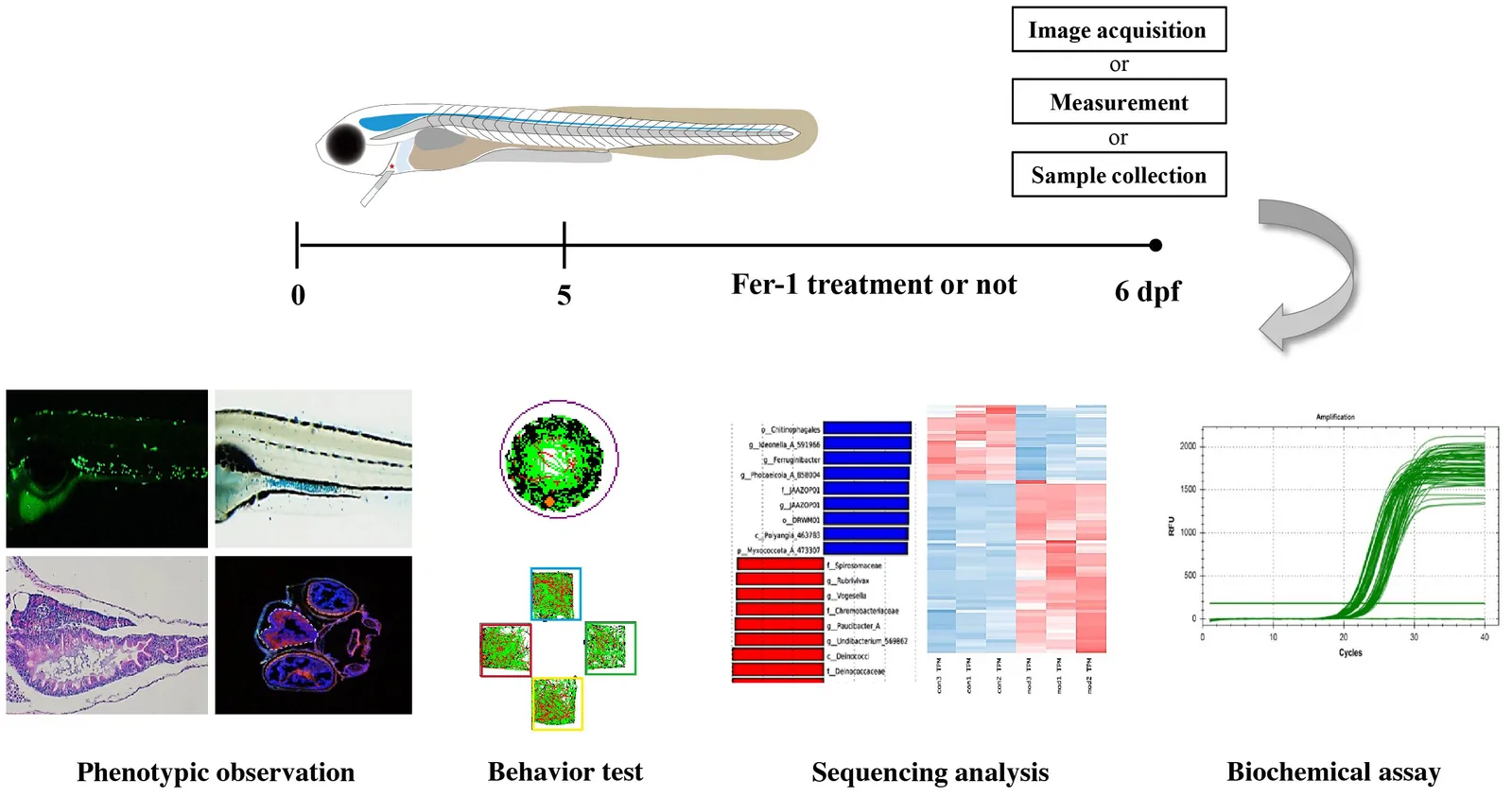
Schematic diagram of LPS-induced larval zebrafish model of sepsis-associated brain dysfunction establishment and drug administration.

## 2 Materials and methods

### 2.1 Animals and maintenance

Adult wild-type AB, transgenic Tg (mpx: EGFP) zebrafish and Tg (gfap:EGFP) zebrafish were provided by Hunter Biotechnology, Inc. (Hangzhou, China). They were maintained in 28°C deionized water (pH 6.5–8.5; conductivity 450–550 μS/cm; hardness 50–100 mg/L CaCO_3_) with a standard 14/10-h light/dark cycle, and the embryos were obtained via natural mating, cultured in embryo media. All animal care and experiments in this study were approved by the Institutional Animal Care and Use Committee (IACUC) of Hunter Biotechnology, Inc. (ethics approval number: IACUC- 2025-8755-25).

### 2.2 SBD model induction

At 5 days post-fertilization (5 dpf), the zebrafish larvae were anesthetized by immersion in 0.16 mg/mL tricaine for 30 s. Then, 10.0 nL of LPS (6.25, 12.5, 25, 50, and 100 mg/mL, Sigma, USA) was microinjected into the peritoneal cavity of each larva. Based on the weight of the larvae (about 0.25 mg/zebrafish larva), the corresponding doses were 0.25, 0.5, 1, 2, and 4 μg/mg. For the control group, the larvae were injected with an equal volume of purified water. After 24 h of treatment at 28°C, the larvae were observed and studied.

### 2.3 Assessment of neutrophil infiltration

The 5 dpf Tg (mpx: EGFP) zebrafish larvae were intraperitoneally administered with 2 μg/mg of LPS to develop the SBD model. After 24 h, 10 larvae of each group were randomly selected for imaging with a fluorescence microscope (AZ10, Nikon, Japan). The NIS-Elements D3.20 advanced image processing software and Image J software were utilized to analyze the total neutrophil amount within delineated regions of interest (ROIs), specifically the entire intestinal tract and the entire brain region of each zebrafish larva. The intestinal ROI was traced from the anterior to the posterior intestine based on anatomical morphology, while the brain ROI was delineated by the cranial contour, excluding the eyes and yolk sac extension. These ROIs were also applied to the subsequent analysis of AO staining and intestinal peristalsis pictures. For ferroptosis inhibitor effect investigation, ferrostatin-1 (Fer-1, MCE, USA) was dissolved in embryo medium. Then, 5-dpf zebrafish larvae treated with LPS were immersed in embryo medium containing Fer-1 and were co-treated for 24 h.

### 2.4 Acridine orange staining

Cell apoptosis was assessed by referring to the reported method with a few adjustments (22). Briefly, 6-dpf alive larvae were stained with 2.5 μg/mL acridine orange (Aladdin, China) at 28°C for 30 min and then washed with fish water three times. Anesthetized with 0.16 mg/mL tricaine, 10 larvae of each group were randomly selected for imaging using a fluorescence microscope, and Image J software was applied to quantify the fluorescence intensity of the apoptotic cells in the brain or intestine ROIs.

### 2.5 Alcian blue staining

At 6 dpf, the larvae were fixed in 4% PFA overnight at 4°C. On the next day, the fixed larvae were washed with acid ethanol (1% HCl/70% ethanol) and incubated in Alcian blue solution (1% Alcian blue, 80% ethanol, and 20% glacial acetic acid) for 3 h at room temperature. Then, the stained larvae were washed three to four times with acid ethanol until all of the blue background was washed away. Moreover, 10 larvae of each group were randomly selected for imaging by using the fluorescence microscope. Goblet cells were predominantly enriched in the mid-to-posterior intestinal segments of zebrafish larvae. The number of goblet cells in the regions were quantified using ImageJ software.

### 2.6 Assessment of reactive oxygen species

For reactive oxygen species (ROS) assessment, 6-dpf zebrafish larvae of each group were applied. Three larvae were put in one well, and 0.5 μg/mL CM-H2DCFDA (Thermo Fisher Scientific, USA) was added for 1 h of incubation. Then, the 96-well microplate was read using a multi-mode microplate reader (SPARK, TECAN, Switzerland) at *λ*_ex_ = 485 nm and *λ*_em_ = 535 nm. The ROS content was calculated by subtracting the fluorescence of the blank controls (fish water without larvae). After detection, three larvae of each group were randomly taken out for fluorescence imaging.

### 2.7 Assessment of behavior impairment

Brain function was assessed through behavior observation, including color preference and locomotor activity. The color-preference behavior test was based on the reported literature (23). At 6 dpf, larvae were individual placed into the central intersection of a four-arm cross-maze (Hunter Biotechnology, China) for 10-min adaptation, and then their further 20-min locomotor activity was recorded and analyzed using a high-throughput 2D behavior analysis system (HT-XW-2D, Hunter Biotechnology, China). The blue-preference index was calculated as the distance traveled in the blue compartment divided by the total distance traveled in the maze.

Locomotor activity was evaluated with a 96-well plate. The larvae were placed separately in each well, and their swimming behavior under a dark environment was continuously monitored for 60 min. Among them, the types of movement distance are classified according to the movement speed: speed <4 mm/s was set as low speed, 4 < speed <20 mm/s was set as middle speed, and 20 mm/s < speed was set as fast speed. The total distance was the sum of the above movement distance, which was used to assess the larvae locomotor activity. These data were also analyzed using the HT-XW-2D software.

### 2.8 Assessment of intestinal peristalsis

At 5 dpf, larvae were treated with LPS for modeling, and then they were exposed to 50 ng/mL Nile Red (Sigma, USA) by static immersion at 28°C for 24 h. The 6-dpf larvae were photographed using a fluorescence microscope for intestinal imaging, and videos of intestinal peristalsis were recorded for typical larvae.

### 2.9 16S rRNA analysis

Whole-body tissues of 120 zebrafish larvae were combined into one sample, with six biological replicates per group, for 16S rRNA sequencing. The DNA of zebrafish was extracted using MagPure Stool DNA KF Kit B (MAGEN, China), and then PCR amplification as well as purification were conducted. Final double-strand library products were denatured to generate the single-strand library products, which further generated the DNA nano ball (DNB) through phi29 amplification and rolling circle amplification. The DNBs were loaded into the patterned nanoarray, and sequencing reads of 250 bp in length were generated using the DNBSEQ-G400 platform (BGI-Shenzhen, China). For data analysis, raw sequencing data were filtered using cutadapt (v.2.6), iTools Fqtools fq (v.0.25), and readfq (v1.0). DADA2 method was used to connect Tags and cluster OUT; alpha diversity measuring the chao index, Shannon index, and Simpson index was calculated using mothur (v.1.31.2); beta diversity analysis to evaluate differences of species complexity was performed using QIIME (v1.80).

### 2.10 Transcriptomic analysis

A total of 30 zebrafish larvae were combined into one sample, with three biological replicates per group, for transcriptome sequencing. According to the manufacturer’s instructions, total RNA was extracted using Trizol (Invitrogen, USA), and RNA quality checking was carried out using Agilent 2100 bioanalyzer (Thermo Fisher Scientific, USA). After the steps of reverse transcription, amplification, and purification, the library was constructed, and single-end 50-bp reads were generated using the BGIseq500 platform (BGI-Shenzhen, China). For data analysis, raw sequencing data were filtered first using SOAPnuke v2.2.1. The acquired clean reads were mapped to the reference genome using HISATA2 v2.2.1. The gene expression levels were calculated using RSEM (v1.3.1), and differential expression was judged with *Q*-value ≤0.05 and |log2 (fold change)| ≥0.585. KEGG enrichment analysis of differentially expressed genes was further performed using the hypergeometric test (Phyper; https://www.kegg.jp/).

### 2.11 Gene expression analysis

For each biological replicate, trunks from approximately 10 larvae or brains from approximately 40 larvae were pooled. Three independent biological replicates were analyzed per group. Total RNA isolation and reverse transcription were successively conducted with RNA extraction kit (Thermo Fisher Scientific, USA) and FastQuant RT Kit (with gDNase; Tiangen, China) according to the manufacturer’s instructions. PowerUp SYBR Green Master Mix (Thermo Fisher Scientific, USA) was used for real-time polymerase chain reaction. The mRNA expression level of the target genes was normalized to β-actin and analyzed using the 2^-ΔΔCT^ method. There were three replicates for each group. The detailed primer sequences are listed in **Supplementary Table S1**.

### 2.12 Detection of blood–brain barrier integrity

This detection referred to the mice model with some adjustments (24). The 5-dpf larvae were treated with LPS for 24 h to develop the SBD zebrafish larvae model. Before imaging, 25 ng of 10-kDa dextran-tetramethylrhodamine (Thermo Fisher Scientific, USA) was injected into the duct of Cuvier of each larva. At 1 h later, 10 larvae of each group were randomly selected for imaging using the fluorescence microscope, and the fluorescence intensity of the brain was quantified using Image J software.

### 2.13 Enzyme-linked immunosorbent assay

The 5-HT and GABA contents were detected using the ELISA assay according to the manufacturer’s instructions (Jonlnbio, China). Approximately 30 larvae were combined into one sample with three biological replicates per group. Briefly, the 5-dpf larvae, after 24 h of LPS treatment, were homogenized on ice with 70 μL PBS using the JXFSTPRP-24L automatic sample rapid grinder (Shanghai Jingxin Industrial Development Co., Ltd., Shanghai, China) and then centrifuged for 10 min (2,500 rpm, 4°C). Subsequently, protein detection and quantification were performed. The enzyme activity level was determined using a multi-mode microplate reader (SPARK, TECAN, Switzerland) at 450 nm.

### 2.14 Acetylcholinesterase enzyme activity detection

The acetylcholinesterase (AchE) enzyme activity was read using alive 6-dpf zebrafish larvae according to the kit’s instruction (AAT Bioquest, USA). Three zebrafish were combined in one well of the 96-well plate with 50 μL E3 media, and then 50 μL AchE working solution was added into each well. After mixing thoroughly, the plates were incubated at 28°C for 60 min, and the fluorescence was subsequently measured using a microplate reader (SPARK, TECAN, Switzerland) at *λ*_ex_ = 490 nm/*λ*_em_ 520 nm.

### 2.15 Histological staining

At 6 dpf, larvae were fixed with 4% paraformaldehyde, embedded in paraffin, and then sectioned into slices (5-μm). Hematoxylin–eosin staining was performed following the manufacturer’s instructions for histopathology observation. Images were captured with a light microscope (Nikon, Japan).

### 2.16 Transmission electron microscopy

Briefly, 6-dpf larvae were sequentially fixed in 2.5% glutaraldehyde and 1% osmic acid. After dehydration and osmosis, the samples were embedded in epoxy resin, and ultraslices (60–80 nm) were stained with uranyl acetate as well as lead citrate. The JEM 1400 transmission electron microscope (JEOL, Japan) was used for sample observation and imaging.

### 2.17 Oxidative stress analysis

To detect the oxidative-stress-related biochemical hallmarks, approximately 30 larvae were combined into one sample with three biological replicates per group. Then, 70 μL PBS was added to each grinding tube for further homogenization with the JXFSTPRP-24L automatic sample rapid grinder. After centrifugation for 10 min (2,500 rpm, 4°C), the supernatant was collected, and protein quantification was conducted. Following the manufacturer’s instructions, we diluted the supernatant and measured the content of MDA (TBA method, cat. no. A003-1-2) as well as 4-HNE (ELISA, cat. no. ml536398V), which were obtained from Nanjing Jiancheng Bioengineering Institute (Nanjing, China).

*In situ* C11-BODIPY staining was performed on 8-μm frozen sections of larvae tissue. The frozen sections were rinsed three times with PBS (5 min for each wash) to remove the OCT compound. A hydrophobic barrier pen was used to delineate the sections on the slides. The sections were then incubated with 2 μM C11-BODIPY (HY-DY1022, MCE) at 37 °C for 30 min in the dark. After three additional washes with PBS (5 min each), the sections were mounted with an antifade mounting medium. Fluorescence images were acquired using a Zeiss LSM 900 confocal microscope (Germany) with dual excitation at 488 and 561 nm. The ratio of mean fluorescence intensity at 510 nm (emission from the oxidized form) to 591 nm (emission from the reduced form) was calculated for each region of interest to evaluate the lipid peroxidation levels (analyzed by Image J). Six biological replicates were performed for statistical analysis.

### 2.18 Immunofluorescence

Immunofluorescence staining of zebrafish sections was performed as follows. The sections were washed with PBS (3 × 5 min), permeabilized with 0.5% Triton X-100 for 20 min, blocked with 3% BSA for 1 h, and incubated with rabbit anti-ZO1 (1:100, 82870-7-RR, Proteintech) and anti-claudin5 (1:100, 29767-1-AP, Proteintech) primary antibodies for 36 h at 4 °C. After the PBS washes, the sections were incubated with Alexa Fluor-conjugated secondary antibodies (1:1,000, ab150077, Abcam) for 2 h at room temperature, followed by ready-to-use DAPI (C1006, Beyotime) staining for 15 min. The sections were mounted and imaged using a Zeiss LSM 900 confocal microscope with a ×10 objective. Mean fluorescence intensity in the brain regions was quantified using Image J, with six biological replicates per group.

### 2.19 Statistical analysis

Statistical analysis was performed using GraphPad Prism 8.0 software (San Diego, CA, USA). All data are presented as mean ± standard deviation. For comparisons between groups, we conducted two-sided unpaired *t*-tests or one-way ANOVA with Tukey’s test, and *p*-value <0.05 was considered statistically significant.

## 3 Results

### 3.1 Microinjecting LPS into zebrafish peritoneal cavity induced intestine inflammation damage

Considering that LPS could cause the death of zebrafish larvae (20, 21), we firstly conducted a maximum tolerated dose exploration. After 5 h of treatment, in the 4 μg/mg group, four dead larvae were observed, and the number of deaths continued to increase in the next 19 h. Finally, there was a mortality rate of 30% after 24 h of exposure to 4 μg/mg LPS, and no deaths were observed in the other groups (**Supplementary Figure S1**). Thus, we chose doses of 0.5, 1, and 2 μg/mg LPS to investigate sepsis-related intestinal phenotypes.

Intestinal inflammation is critical to sepsis occurrence and development. The massive release of inflammatory factors can damage the intestinal functional cells and the intestinal barrier mucosa, resulting in altered intestinal motility and barrier dysfunction (25). When larvae were treated with different doses of LPS, compared with the control group, the number of intestinal neutrophils and the expression of inflammatory genes (*IL6*, *IL1β*, *IFNγ*, and *TNFα*) were dose-dependently enhanced (**Figures 2A,D**), suggesting the occurrence of intestinal inflammation. Goblet cells are important functional cells in the intestine. It not only secretes mucus to stabilize intestinal mucosal barrier but also participates in the regulation of the intestinal immune system. In the LPS-treated larval group, the number of Alcian blue-marked goblet cells were significantly reduced (**Figure 2B**). Simultaneously, after 24 h of exposure to Nile Red after LPS treatment, intestinal peristalsis was enhanced, and the remaining intestinal fluorescence was reduced in a dose-dependent manner (**Figure 2C** and **Supplementary Videos S1**–**S4**). Furthermore, the AO staining and pathological staining results showed an elevated number of apoptotic cells and reduced villi and folds in the larval intestine in the 2 μg/mg LPS group compared with the control group (**Supplementary Figure S2**; **Figure 3A**).

**Figure 2:**
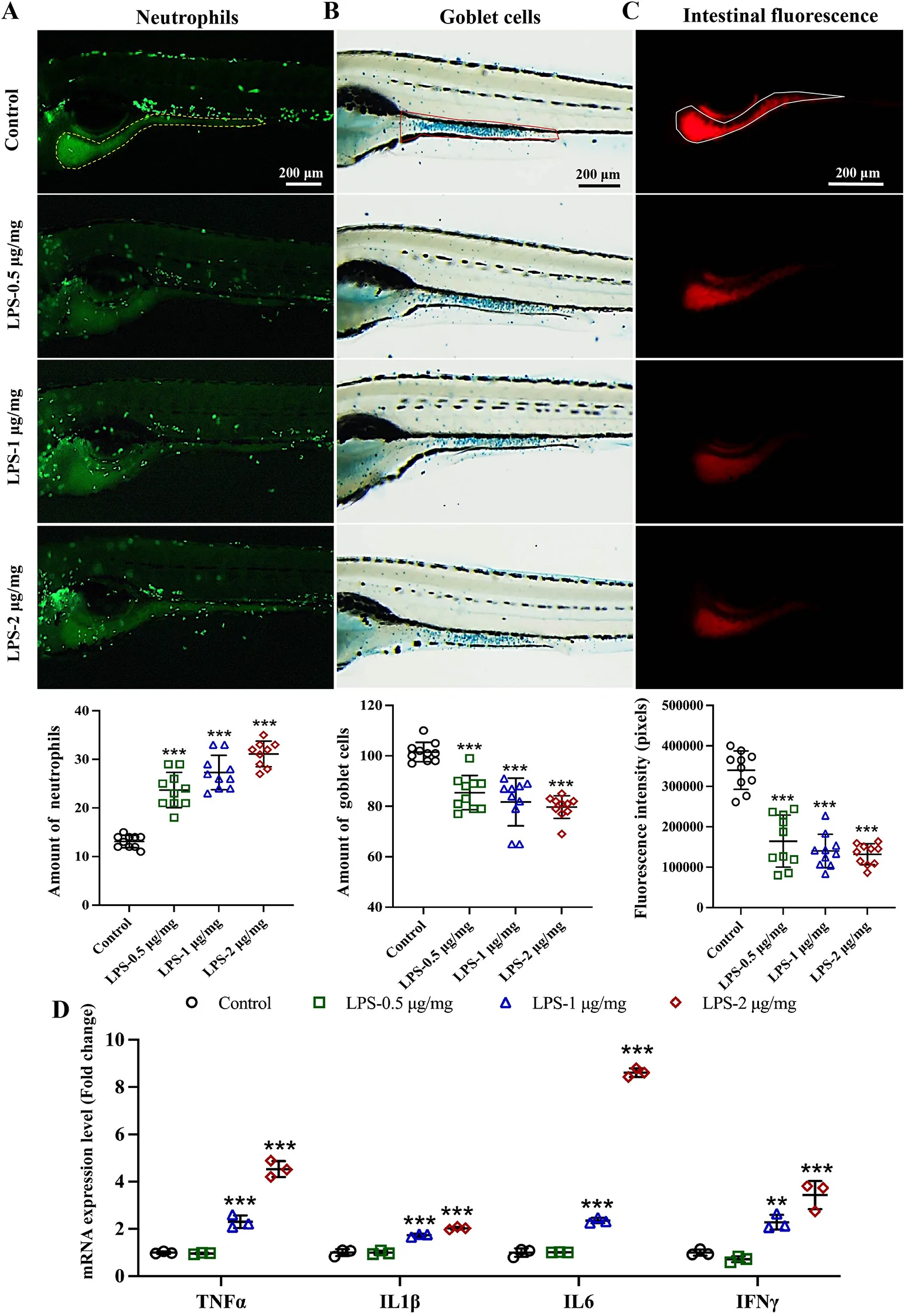
Intraperitoneal administration of LPS into zebrafish induced intestine inflammation damage. Representative images and quantitative analysis of intestinal neutrophil number **(A)**, goblet cell number **(B)**, and Nile Red fluorescence **(C)** within the indicated region of interest of each larva. Colored boxes demarcate the regions of interest for statistical analysis of each index. Yellow arrow: intestinal neutrophils; *n* = 10 biological replicates. **(D)** Relative mRNA levels of inflammatory genes. *n* = 3 biological replicates. Data are presented as mean ± SD. Compared with the control group: **p < 0.01, ***p < 0.001.

**Figure 3:**
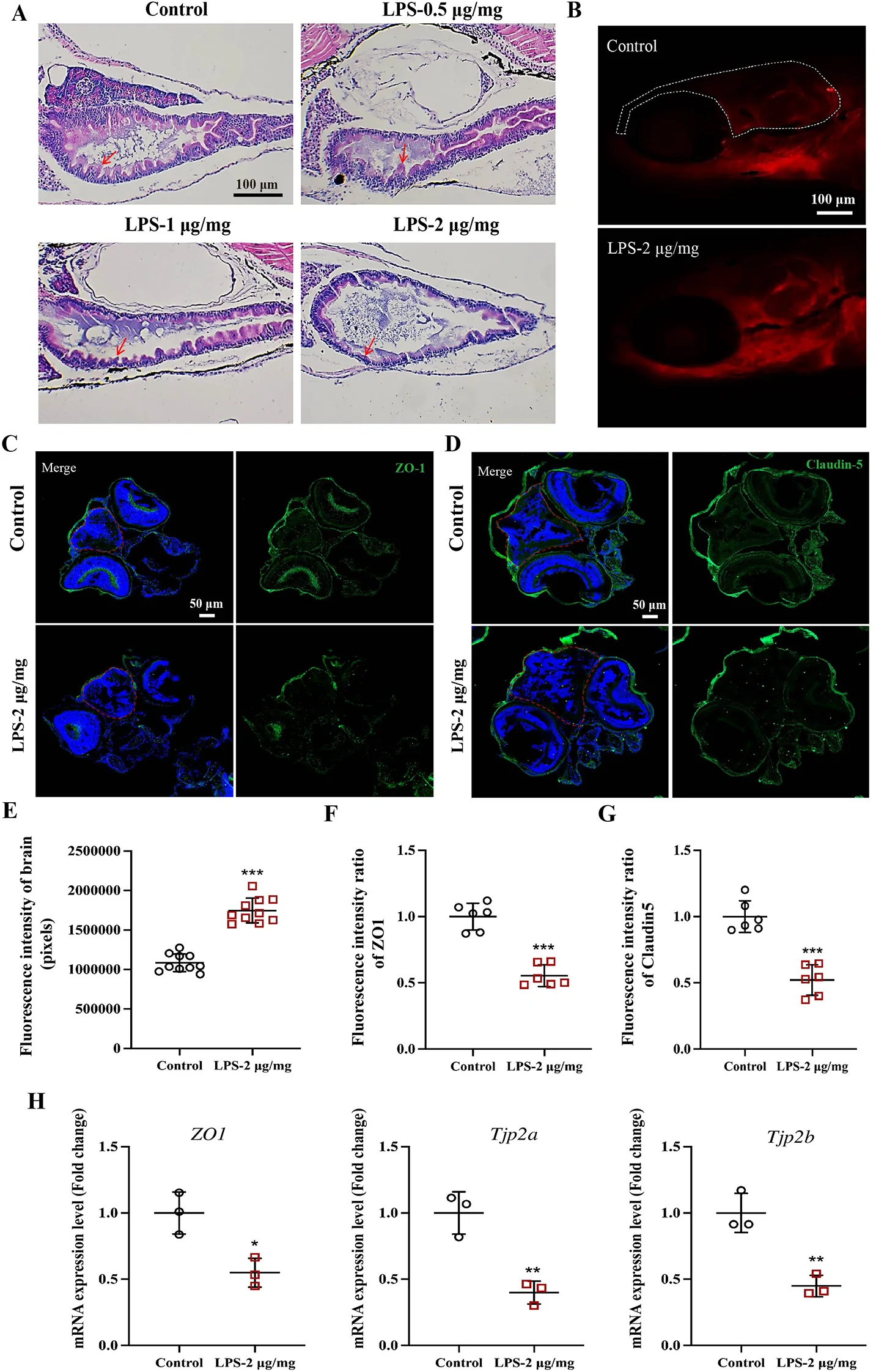
Intraperitoneal administration of LPS influenced intestinal structure and blood–brain barrier integrity. Representative images of histopathological sections of zebrafish intestines **(A)** and dextran-tetramethylrhodamine leakage in zebrafish brain **(B)**. Red arrows indicate intestinal folds and villi. White box: dextran-tetramethylrhodamine leakage analysis area. Representative immunofluorescent images and quantitative analysis of ZO1 **(C, F)** and claudin5 **(D, G)**; red boxes demarcate the brain regions of interest for statistical analysis of markers; *n* = 6 biological replicates. **(E)** Fluorescence quantitative analysis of **(B)**, *n* = 10 biological replicates. **(H)** Relative mRNA levels of barrier-related genes, *n* = 3 biological replicates. Data are presented as mean ± SD. Compared with the control group: *p < 0.05, **p < 0.01, ***p < 0.001.

These findings show that intraperitoneal LPS administration induced intestinal inflammation, structural injury, and altered intestinal function. Because the dose of 2 μg/mg LPS induced obvious changes in all of the abovementioned phenotypes, this dose was applied for further phenotype observation.

### 3.2 Intraperitoneal administration of LPS influenced blood–brain barrier permeability

During the development of SAE, continuous inflammation as well as oxidative stress can downregulate tight junction proteins and subsequently increase BBB permeability, which is critical to brain dysfunction (7). Thus, before investigating the effects of LPS intraperitoneal administration on zebrafish brain, we conducted an observation on blood–brain barrier integrity. It was found that in the LPS-treated group of zebrafish, the leakage of 10 kDa fluorophore to the brain was obviously increased (**Figures 3B, E**). To validate BBB integrity at the protein level, we performed immunofluorescence staining for ZO1 and claudin5 on larvae brain sections. As presented in **Figures 3C, D**, in the control group, both proteins displayed continuous linear signals surrounding the brains, indicating intact tight-junction belts. In contrast, the LPS-treated group exhibited a significant reduction in mean fluorescence intensity (**Figures 3F, G**) and pronounced discontinuity of the junctional signals, with fragmented and gapped distribution. These results demonstrate that LPS compromised BBB structural integrity at the protein level, corroborating the increased permeability observed in dextran leakage assays. Additionally, the expression of brain-barrier-related genes, including *ZO1*, *Tjp2a*, and *Tjp2b*, was significantly downregulated with LPS treatment (**Figure 3H**). These data suggested that there was also increased BBB permeability in the developed zebrafish model, in consistence with SAE mice model.

### 3.3 Intraperitoneal LPS administration induced neuroinflammatory, neurochemical, and behavioral alterations

It is well-known that brain inflammation and abnormal behaviors are important pathological features of SAE. Utilizing the transgenic fluorescent zebrafish to observed inflammation change in zebrafish brain, it was found that in the LPS group the number of infiltrated neutrophils and the fluorescence of gfap-marked astrocytes were significantly more than those of the control group (**Figure 4A**; **Supplementary Figure S3**), suggesting the activation of neuroinflammation. The AO staining results showed an obvious increase in the number of apoptotic cells in the brains of LPS-treated larvae (**Figure 4B**). Simultaneously, based on the natural movement habits and blue color preference of zebrafish, neurobehavioral experiments were conducted to explore the impact of this model on brain function. As shown in **Figure 4C**, the larvae in the 2-μg/mg-LPS group exhibited shorter movement distances under medium and high speeds, and the overall movement distance was correspondingly reduced, suggesting that their locomotivity had been affected. The blue-preference index was significantly reduced in LPS-treated larvae (**Figure 4D**), indicating altered color preference or exploratory behavior; however, this reduction was likely influenced, at least in part, by the marked locomotor hypoactivity observed after LPS exposure. To assess neurochemical alterations associated with the behavioral phenotype, we measured the 5-HT and GABA levels and AChE activity as focused readouts of serotonergic, GABAergic, and cholinergic-related regulation (26–28). LPS exposure significantly increased the 5-HT and GABA levels and AChE activity (**Figures 4E–G**), indicating altered neurochemical homeostasis.

**Figure 4:**
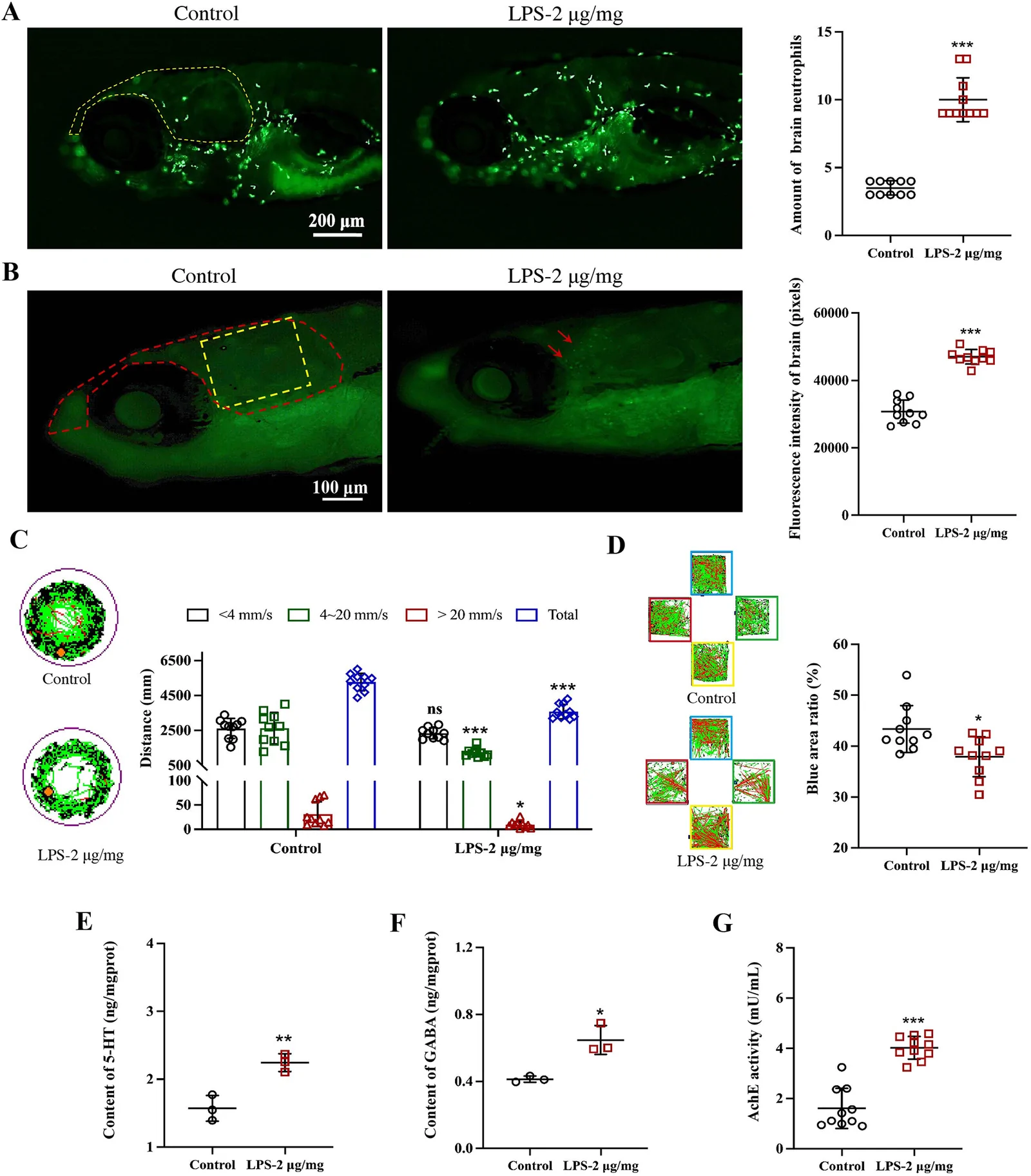
Intraperitoneal administration of LPS induced brain inflammation dysfunction. Representative images and quantitative analysis of brain neutrophils **(A)** and apoptosis cells **(B)**. Colored boxes demarcate the regions of interest for statistical analysis of corresponding index. Red arrows indicate apoptotic cells; *n* = 10 biological replicates. Representative swimming trajectories and quantitative analysis in movement test **(C)** and cross-maze test **(D)**, *n* = 10 biological replicates. Neurotransmitter content of 5-HT **(E)** and GABA **(F)**, *n* = 3 biological replicates. **(G)** Enzyme activity of AchE, *n* = 10 biological replicates. Data are presented as mean ± SD. Compared with the control group: *p < 0.05, **p < 0.01, ***p < 0.001.

The abovementioned data indicated that the developed model not only simulated intestinal damage but also led to brain inflammation as well as dysfunction.

### 3.4 LPS exposure altered the larva-associated microbial community and ferroptosis-related signaling

To characterize LPS-associated changes in larval microbial communities, 16S rRNA sequencing was performed in the control and 2-μg/mg-LPS group. There was a total of 793 OTUs in the data, of which approximately 25.8% were common among the two groups (**Figure 5A**). Principal component analysis (PCA) showed clear separation between the two groups (**Figure 5B**). The Shannon and Simpson indices differed significantly between groups, whereas the Chao richness index did not (**Figure 5C**), indicating altered community diversity without a detectable change in richness. According to the phylum-level analysis, compared with the control group, the relative abundance of the dominant bacterial phylum *Proteobacteria* in LPS-treated zebrafish increased by approximately 39.8% (**Figure 5D**). Moreover, the genus-level analysis showed that LPS treatment obviously enhanced the relative abundance of *Aeromonas* (belonging to the phylum *Proteobacteria*) by 137.7% (**Figure 5E**). Because DNA was extracted from whole larvae and taxonomic classification was limited to the genus level, the increased relative abundance of *Aeromonas* was interpreted as part of the overall LPS-associated shift in the larva-associated microbial community.

**Figure 5:**
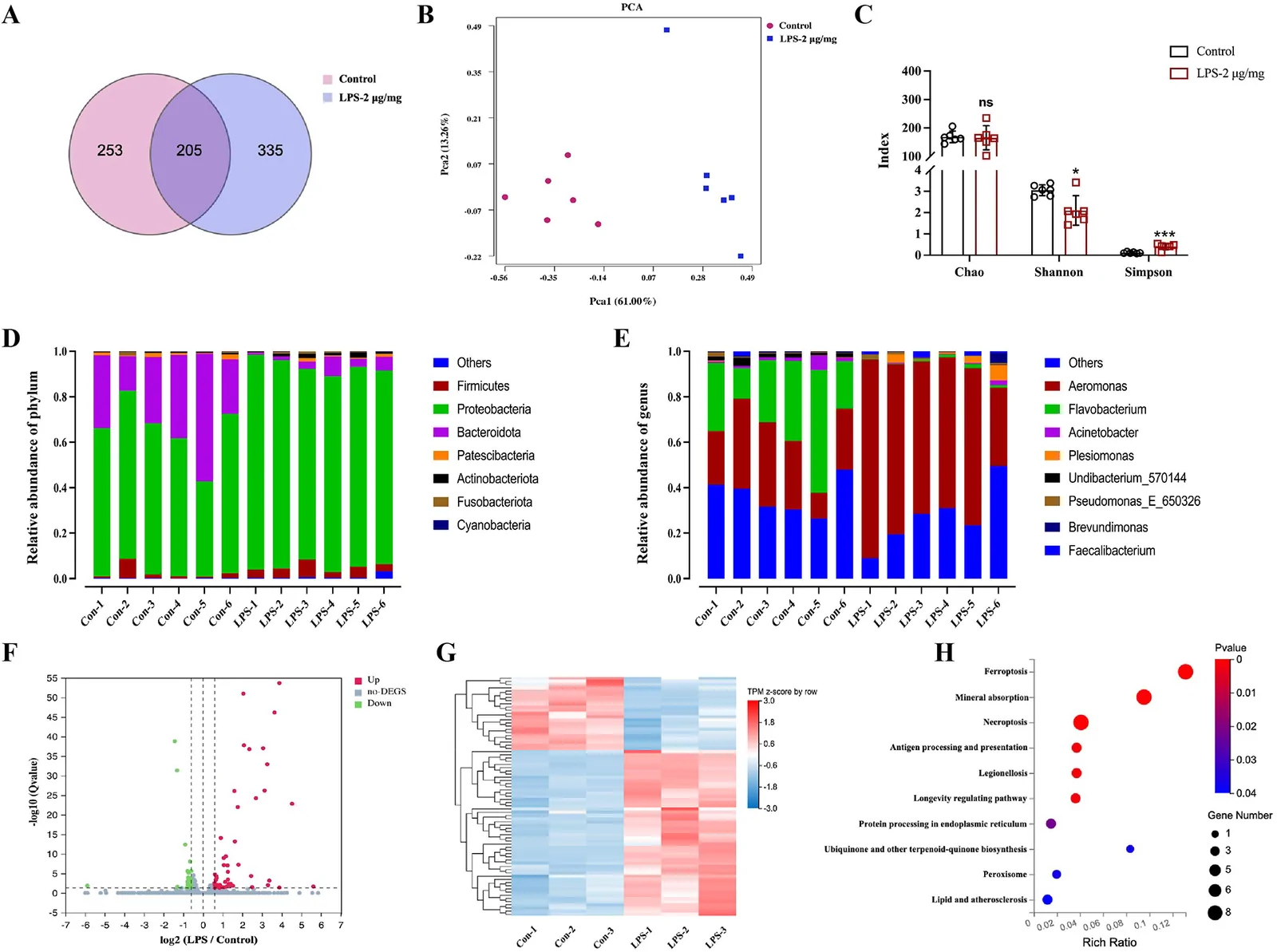
Analysis of *16S* sequencing and transcriptome. **(A)** Venn plot on OUT distribution, **(B)** PCA score plot, **(C)** microbial diversity; microbiota composition at the phylum level **(D)** and genus level **(E)**, *n* = 6 biological replicates. Volcano plot on DEGs distribution **(F)**. Hierarchical clustering heatmap of DEGs **(G)**. KEGG pathways’ analysis of DEGs **(H)**, *n* = 3 biological replicates. Data are presented as mean ± SD. Compared with the control group: *p < 0.05, ***p < 0.001; ns, no significant difference.

Whole-larva transcriptomic profiling was also performed. Compared with the control group, 75 differentially expressed genes were identified using an FDR-adjusted Q-value ≤0.05 and |log2(fold change)| ≥0.585, with significant enrichment of ferroptosis-related signaling (**Figures 5F–H**). Ferroptosis had been reported to be involved in sepsis and sepsis-related organ damage. During sepsis, iron metabolism was dysregulated, which induced the activation of inflammatory pathway as well as ferroptosis, further promoting the release of damage-associated molecular patterns or oxidation products (29). Based on the finding of the transcriptome analysis, we first monitor the level of oxidative stress, and it was observed that the total ROS fluorescence intensity in the model group was significantly increased. Specifically, the ROS fluorescence in both the intestine and the brain was stronger than that in the control group (**Figures 6A,B**). Upon detection of ferroptosis-related gene expression levels, the brain of zebrafish exhibited downregulated *Gpx4* and *Slc7a11* and upregulated *Acsl4* and *Nox1* when exposed to LPS, supporting ferroptosis-related alterations (**Figure 6C**). The electron microscopy results showed that in the brain tissue of model zebrafish, the mitochondria in the cytoplasm were slightly swollen, the electron density decreased, and, in some cases, the cristae were sparse or broken (**Figure 6D**).

**Figure 6:**
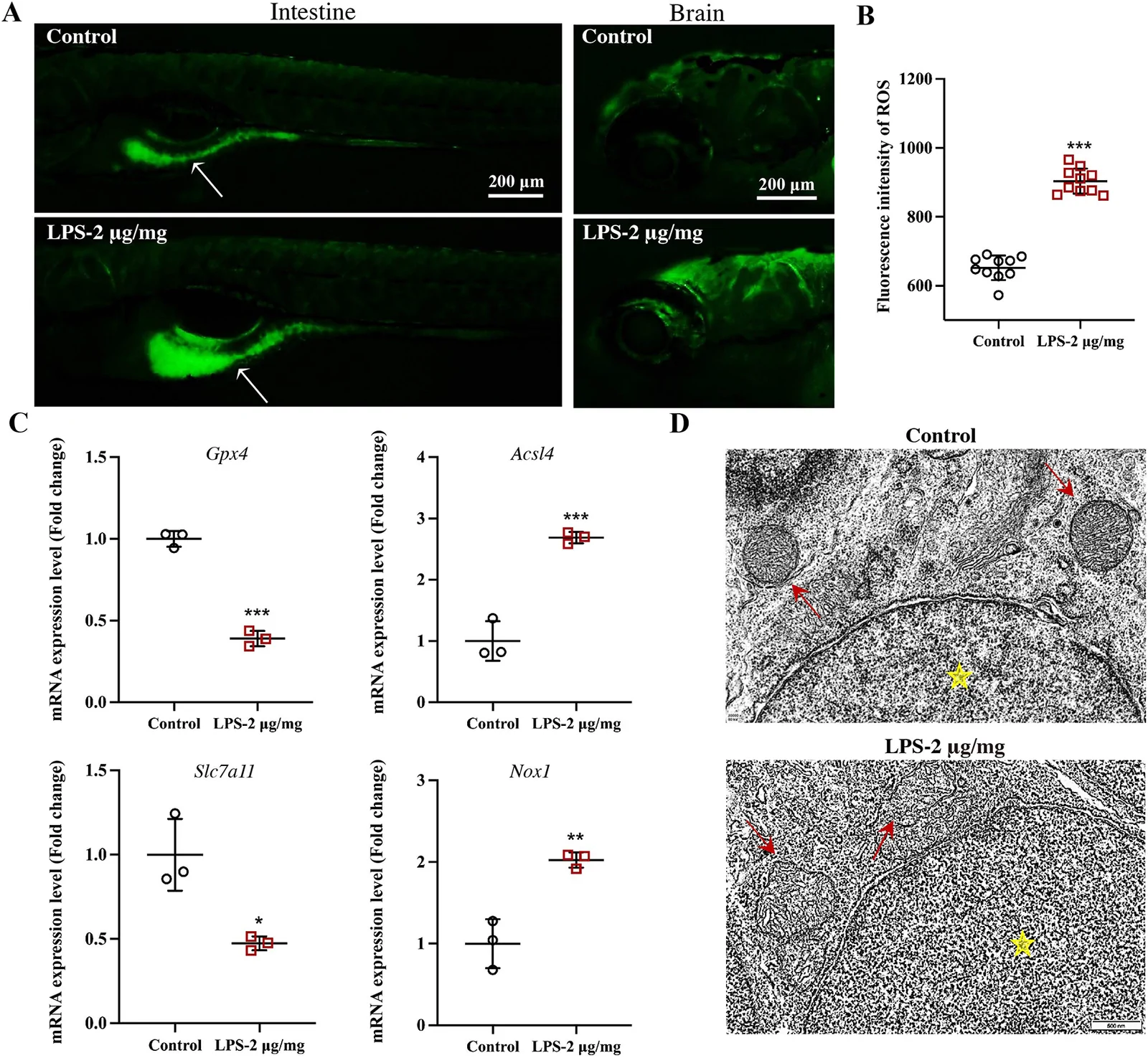
Ferroptosis was involved in SBD zebrafish model. Representative images and quantitative analysis of ROS level **(A, B)**. White arrows indicate zebrafish intestine; *n* = 10. Relative mRNA levels of ferroptosis-related genes **(C)**, *n* = 3 biological replicates. Representative brain electron microscopy images for zebrafish **(D)**. Red arrows indicate mitochondria; yellow stars indicate nucleus. Data are presented as mean ± SD. Compared with the control group: *p < 0.05, **p < 0.01, ***p < 0.001.

The sequencing analyses together showed that LPS exposure was accompanied by changes in the larva-associated microbial community and a prominent ferroptosis-related transcriptional response.

### 3.5 Ferroptosis inhibitors exhibited improved effect in the developed SBD model

To investigate the drug screening potential of the developed SBD zebrafish model, based on the transcriptome result, we further validated the effect using the ferroptosis inhibitor Fer-1. Because the maximum non-lethal concentration of Fer-1 in SBD zebrafish model was 12.5 μM, it was chosen as the highest concentration for efficacy evaluation. The results showed that there was a concentration-dependent reduction in neutrophil infiltration in the intestine and brain with Fer-1 treatment (**Figures 7A–D**). Moreover, 12.5 μM Fer-1 significantly increased the overall movement distance, effectively improving LPS-induced reductions in locomotor activity (**Supplementary Figure S4A**). Moreover, the result of AO staining showed that 12.5 μM Fer-1 could decrease cell apoptosis in larvae brain (**Figure 7E**). Upon further examination of the effects of Fer-1 treatment on the biochemical hallmarks of ferroptosis in larvae, the results revealed that Fer-1 intervention significantly reduced ROS accumulation and decreased the levels of MDA and 4-HNE (**Figures 7F–H**). *In situ* C11-BODIPY staining of tissue sections was performed to assess lipid peroxidation within the larvae brain. The results demonstrated that Fer-1 diminished the production of oxidized lipids, as evidenced by a reduced ratio of oxidized to reduced lipid species, thereby effectively ameliorating lipid peroxidation (**Figure 7I**; **Supplementary Figure S4B**).

**Figure 7:**
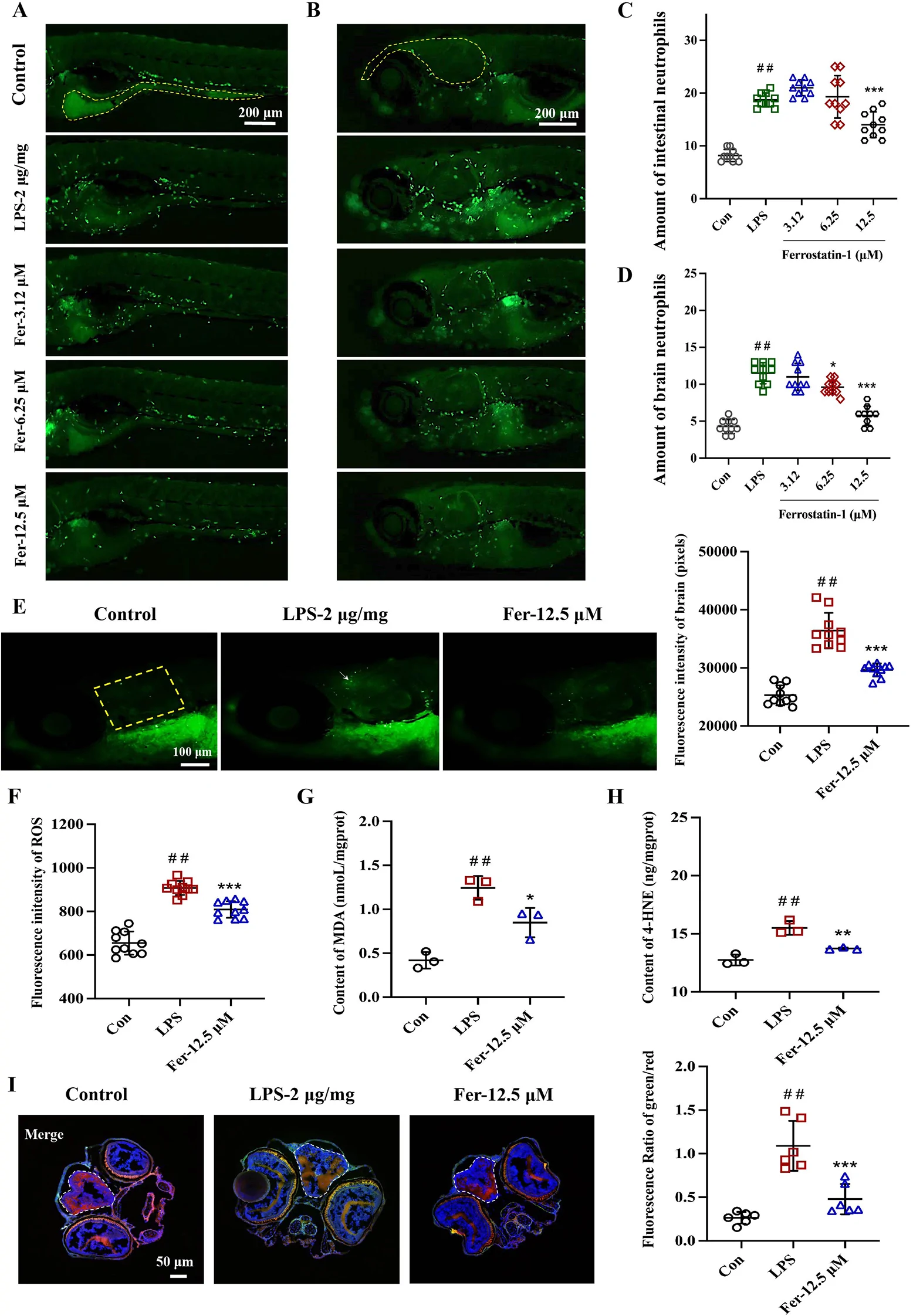
Improvement of Fer-1 on developed SBD zebrafish larvae model. Representative images and quantitative analysis of intestine neutrophil number **(A, C)**, brain neutrophil number **(B, D)**, and brain apoptotic cell number **(E)**; yellow boxes demarcate the regions of interest for statistical analysis of each index; *n* = 10 biological replicates. Quantitative determination of ROS level **(F)**, MDA content **(G)**, and 4-HNE content **(H)**. For ROS detection, *n* = 10 biological replicates; biochemical index detection, *n* = 3 biological replicates. Representative merged images and quantitative analysis of lipid peroxidation staining with C11-BODIPY **(I)**. White boxes demarcate the brain regions of interest for statistical analysis of lipid peroxidation; oxidized lipids were marked with green fluorescence, and reduced lipid species were marked with red fluorescence; *n* = 6 biological replicates. Data are presented as mean ± SD. Compared with the control group: #*p* < 0.01, ##*p* < 0.001. Compared with the LPS group: *p < 0.05, ***p < 0.001.

Furthermore, we explored the regulation of Fer-1 at the molecular level. As shown in **Figure 8**, treated with 12.5 μM Fer-1, the zebrafish exhibited downregulated brain inflammation-related genes (*IL6*, *IL1β*, *IFNγ*, and *TNFα*), upregulated brain barrier-related genes (*Tjp2a* and *Tjp2b*), and downregulated brain apoptosis-related genes (*Bax*, *Bcl2*, *Casp3*, and *Casp9*). For the brain ferroptosis-related genes, the expression of *Gpx4* and *Acsl4* was increased and decreased, respectively (**Figure 8C**). These data indicated that the ferroptosis inhibitor of Fer-1 presented improvement potential in SBD. Combined with the advantage of high throughput, the developed SBD zebrafish larvae model was expected to be beneficial for SAE drug discovery.

**Figure 8:**
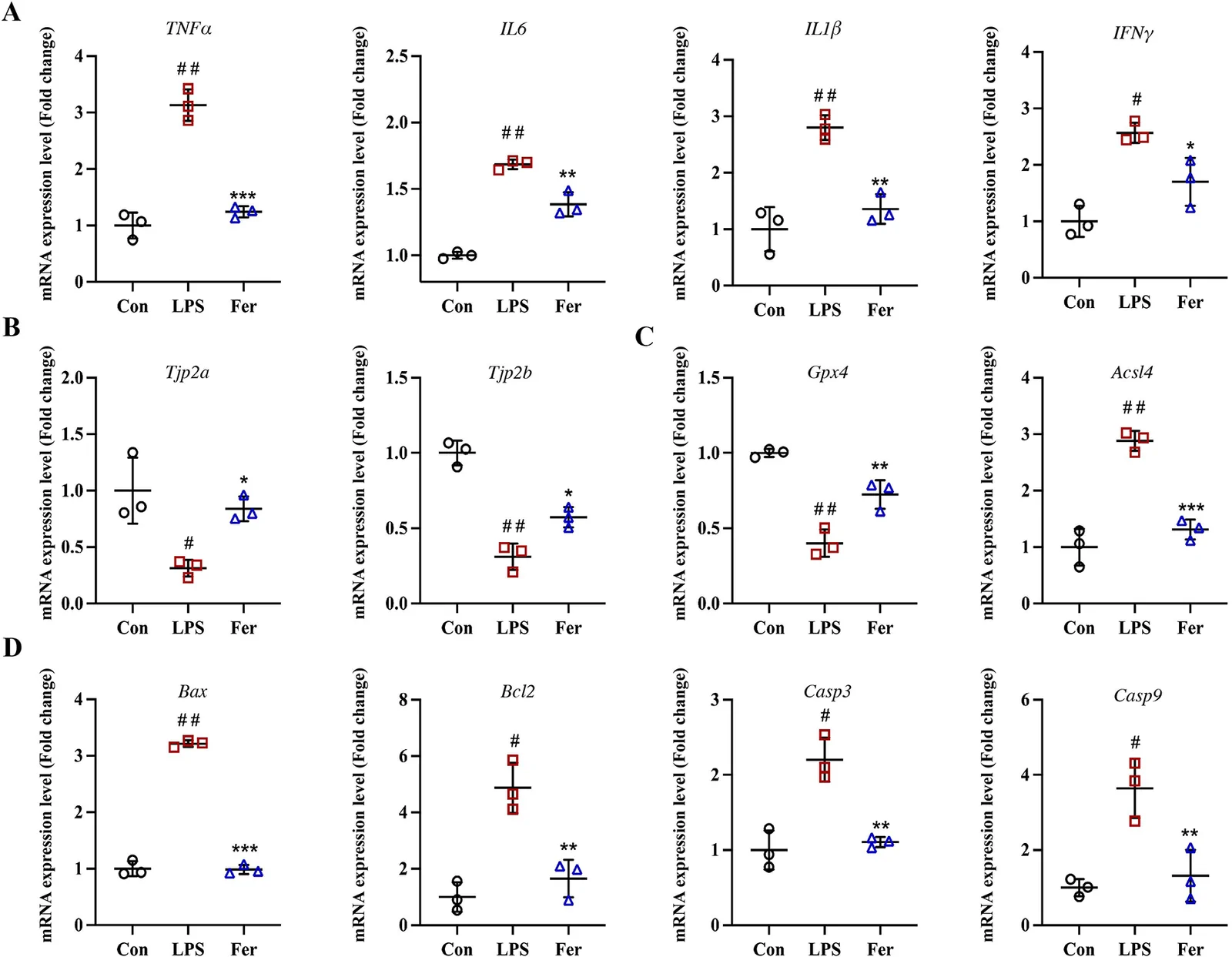
Regulation of Fer-1 on relative mRNA levels of brain inflammatory genes **(A)**, barrier-related genes **(B)**, ferroptosis-related genes **(C)**, and apoptosis-related genes **(D)** with SBD larvae model, *n* = 3 biological replicates. Data are presented as mean ± SD. Compared with the control group: #*p* < 0.01, ##*p* < 0.001. Compared with the LPS group: *p < 0.05, **p < 0.01, ***p < 0.001.

## 4 Discussion

Effective treatments for SAE remain limited because of its heterogeneous onset and complex pathophysiology. In this study, we established a larval zebrafish model of sepsis-associated brain dysfunction (SBD) through the intraperitoneal microinjection of LPS. Compared with previous zebrafish sepsis models based on LPS exposure in embryo medium (20, 21), the administration of a defined intraperitoneal dose improves dosing consistency. The larval SBD model displayed sepsis-relevant intestinal injury, increased BBB permeability, neuroinflammation, behavioral alterations, and brain cell apoptosis. Given its short experimental cycle, low sample requirement, optical accessibility, and suitability for higher-throughput analysis, this model may provide a useful platform for phenotypic drug screening in SBD.

The 5-day-post-fertilization larvae were selected because their nervous, circulatory, and digestive systems are sufficiently developed to support inflammatory, vascular, and behavioral assessments (30). Because intraperitoneal microinjection provides direct LPS exposure, we first performed a dose tolerance assessment. Mortality was initially observed 5 h after the administration of 4 μg/mg LPS and reached 30% at 24 h, whereas no mortality was observed at the lower doses. Therefore, 0.5, 1, and 2 μg/mg LPS doses were selected for subsequent phenotypic characterization. LPS exposure produced dose-dependent intestinal neutrophil accumulation, inflammatory-gene expression, altered intestinal motility, goblet-cell loss, and structural injury. The effective mass-normalized doses were higher than those commonly used in rodent LPS models (15). This difference is consistent with the relatively low sensitivity of zebrafish to LPS, which has been associated with species-specific differences in LPS recognition and signaling (31, 32).

In addition to intestinal dysfunction, central nervous system alterations are important features of SAE and may involve BBB dysfunction, neuroinflammation, astrocytic and microglial activation, neurotransmitter imbalance, oxidative stress, and neuronal cell death (5, 33, 34). Consistent with these features, the larval SBD model showed increased cerebral dextran leakage, brain neutrophil infiltration, enhanced Tg (gfap:EGFP) fluorescence, brain cell apoptosis, reduced locomotor activity, and altered color-preference/exploratory behavior. Because LPS also markedly reduced the total swimming distance, locomotor hypoactivity likely contributed to the lower blue-preference index; therefore, this finding was interpreted as altered color-preference/exploratory behavior rather than direct evidence of cognitive impairment. Future studies may apply covariance analysis using locomotor distance as a covariate to better distinguish motor impairment from exploratory decline. Microabscesses, which have occasionally been reported in severe cases of SAE (2), were not observed, suggesting that the present model predominantly reproduces relatively mild brain-dysfunction-related phenotypes. The 5-HT, GABA, and AChE measurements were selected as focused neurochemical readouts because serotonergic and GABAergic signaling can influence neural activity and locomotor behavior in larval zebrafish (26, 27), whereas cholinergic signaling participates in inflammatory regulation during endotoxemia (28). The observed changes therefore indicate neurochemical alterations associated with the LPS-induced behavioral phenotype.

Increasing evidence indicates that the gut microbiota participates in neurodevelopment and neurological regulation through the gut–brain axis. Alterations in microbial composition and function have also been reported in SAE (35–38). In the present study, LPS exposure was associated with an increased relative abundance of *Aeromonas*. LPS-induced oxidative stress and mucosal barrier breakdown may reshape the local intestinal microenvironment. Under inflammatory conditions, the availability of respiratory electron acceptors, such as nitrate, can increase, which may selectively favor facultative anaerobic Proteobacteria. In addition, disruption of the mucus layer may alter the spatial organization and niche availability of *Aeromonas* (39, 40). These changes could together create a selective microenvironment that supports the expansion of facultative proteobacteria, including *Aeromonas*. The observed increase in the relative abundance of *Aeromonas* is therefore consistent with inflammation-associated dysbiosis. However, this finding should not be interpreted as direct evidence of pathogenic bacterial expansion or brain invasion. *Aeromonas* species are present in the normal zebrafish microbiota (41), and their effects are species- and strain-dependent: some *A. veronii* strains promote host–microbe mutualism and limit excessive intestinal inflammation (42), whereas other isolates can induce pro-inflammatory cytokine expression (43). Moreover, because LPS itself directly induces inflammatory cytokines, the present results cannot determine whether the increase in *Aeromonas* contributed to the observed *IL6, IL1β, IFNγ*, or *TNFα* changes. Therefore, we interpret the increase in *Aeromonas* as an association within the altered larva-associated microbial community. Species-level identification, spatial localization, and bacterial colonization or depletion experiments are required to determine its causal contribution. Simultaneously, with the analysis of transcriptome sequencing, ferroptosis was significantly regulated, which recently had been regarded as a potential therapeutic target for SAE. Validation with the inhibitor of ferroptosis showed that it exhibited an effective improvement in SBD, indicating the drug screening potential of the established zebrafish larvae model. Inflammatory pathways did not reach the adjusted significance threshold in the KEGG over-representation analysis, although targeted qPCR detected significant changes in *IL6, IL1β, IFNγ*, and *TNFα*. This apparent difference does not necessarily indicate an absence of inflammation. Over-representation analysis depends on the number of genes passing the differential-expression cutoff and may have limited sensitivity when transcriptional changes are modest or distributed across multiple pathways (44, 45). Moreover, in teleost larvae, the early cytokine response may already be subsiding by 24 h, whereas ferroptosis-related oxidative injury may remain prominent; thus, bulk whole-larva RNA sequencing at this single time point may underrepresent transient or cell-specific inflammatory signals that remain detectable by targeted qPCR. Therefore, the qPCR findings are interpreted as targeted evidence of inflammatory gene regulation, whereas ferroptosis is described as a prominent, but not exclusive, transcriptomic response under the present analytical conditions. Future time-course and cell-resolved analyses will be required to define the temporal and cellular inflammatory responses. Several limitations should be considered. First, the principal phenotypic and molecular endpoints were assessed at a single 24-h time point, precluding assessment of temporal changes in the cytokine and ferroptosis responses. Second, whole-larva 16S rRNA sequencing cannot spatially distinguish intestinal, skin-associated, and other larva-associated microorganisms. Similarly, bulk whole-larva transcriptomic profiling lacks tissue and cell-type resolution. Third, reduced locomotor activity may have influenced performance in the color-preference assay. Finally, the larval LPS model does not reproduce all clinical features of SAE, and cerebral ischemic changes and perivascular edema were not assessed in the present study. Time-course, spatially resolved, and tissue- or cell-specific analyses will help define the scope and applicability of this model more precisely.

## 5 Conclusion

In this study, we established an intraperitoneal LPS-induced larval zebrafish model of sepsis-associated brain dysfunction. The model displayed intestinal inflammation and injury, increased BBB permeability, neuroinflammation, reduced locomotor activity, altered color-preference/exploratory behavior, neurochemical alterations, and brain cell apoptosis. Omics analyses further identified LPS-associated changes in the larva-associated microbial community and ferroptosis-related signaling. Fer-1 partially improved several inflammatory, behavioral, oxidative stress, and cell-death-related phenotypes. Together with the advantages of larval zebrafish for rapid and visualized assessment, this model may provide a useful platform for phenotypic drug screening and further investigation of sepsis-associated brain dysfunction.

## Data Availability

The datasets presented in this study can be found in online repositories. The names of the repository/repositories and accession number(s) can be found below: National Genomics Data Center (NGDC) (https://ngdc.cncb.ac.cn/bioproject/browse/PRJCA070832), PRJCA070832.

## Supplementary material

Supplementary Video 1 Control.

Supplementary Video 2 LPS-0.5 μg/mg.

Supplementary Video 3 LPS-1 μg/mg.

Supplementary Video 4 LPS-2 μg/mg.
