# Supplementary material for: Establishing a novel sepsis-associated brain dysfunction model in zebrafish larvae
Source: Front Immunol. 2026 Oct 29;17:1862109. doi: 10.3389/fimmu.2026.1862109 (PMC13754749; doi:10.3389/fimmu.2026.1862109)
Supplement: Supplementary file 1 [file DataSheet1.docx]

**Supplementary Information**

**Establishing a novel sepsis-associated brain dysfunction model in zebrafish larvae**

Siqi Wu^a,b,c,1^, Tao Zhang^b,1^, Shengya Guo^b^, Zhongyan Li^b^, Xujie Pan^b^, Yanfeng Huang^b^, Lingling He^b^, Yong Zhang^b,c,d,^ *, Ming Jiang^a^ *

^a^ Center for Genetic Medicine and Department of Otolaryngology-Head and Neck Surgery, The Fourth Affiliated Hospital, Zhejiang University School of Medicine, Hangzhou, 310030, Zhejiang, China.

^b^ Hunter BioInsight Technology, Inc., Hangzhou 310051, China.

^c^ Hunter Biotechnology, Inc., Hangzhou 310051, China.

^d^ State Key Laboratory of Natural Medicines, Department of Pharmacology, School of Pharmacy, China Pharmaceutical University, Nanjing 211198, China.

**Contents**

**Figure S1:** The investigation of MTD for LPS intraperitoneal administration.  S-2

**Figure S2:** The cell apoptosis of intestine for SBD model with AO staining. S-3

**Figure S3:** The influence on brain area and amount of astrocytes of SBD model. S-4

**Figure S4:** The improvement of Fer-1 on developed SBD zebrafish larvae model. S-5

**Table S1:** Primers used in qPCR. S-6

**Figure S1** The survival of 5 dpf larvae when exposed to LPS 24 h with intraperitoneal administration (n=30 biological replicates).

**Figure S2** The cell apoptosis of intestine for SBD model with AO staining. The area within the yellow frame was the analyzed intestine area; **^***^***p* < 0.001 compared to the control group, n=10 biological replicates.

**Figure S3** The influence on brain area and amount of astrocytes for SBD model. The area within the red frame was astrocytes analyzed area; ns: no significant difference; **^***^***p* < 0.001 compared to the control group, n=10 biological replicates.

**Figure S4** The improvement of Fer-1 on developed SBD zebrafish larvae model. Representative images and quantitative analysis of movement test (A) and lipid peroxidation staining with C11-BODIPY. The behavior test n=10 biological replicates; immunofluorescence statistical analysis n=6 biological replicates. Data are presented as mean ± SD. Compared with the control group: #*p* < 0.01, ##*p* < 0.001. Compared with the LPS group: **p* < 0.05, ****p* < 0.001.

.

**Table S1** Primers used in qPCR.

| Gene | Forward Primer (5’ > 3’) | Reverse Primer (5’ > 3’) |
| --- | --- | --- |
| *ZO1* | ACGGAGTCTGTGGAGAAGGTGAG | GGTGAGTTTGGGCAGGGATGTG |
| *IFNγ* | GCCTGGGGAGTATGTTTGCT | CAGGAAGATGGGGTGTGCAT |
| *Tjp2a* | GTGAGGCTAAACACCATTC | TGCTTTGACAGACCCAGA |
| *Tjp2b* | TGATGGCTGTTAGGTTTCTG | GGGTAATCTGAGTCTGGGTC |
| *Gpx4* | GAGGTTTACGCATCCTGGCT | GGCTGATCCTTCAGCCACTT |
| *SLC7A11* | TGTGGGAATGTCACTGGTGG | ACGCCTCCAGAATGTACGTG |
| *Nox1* | TTTAGGGTCTGCATTGGCTTGGG | GCAGCAGAGACAGCAGGTTACG |
| *Acsl4* | AGCGGACTGGCAGCACTAGG | GAAGCAGGCTTGAGCGGTGATC |
| *Bcl2* | AGGAAAATGGAGGTTGGGATG | TGTTAGGTATGAAAACGGGTGGA |
| *Bax* | GGCTATTTCAACCAGGGTTCC | TGCGAATCACCAATGCTGT |
| *Casp3* | CCGCTGCCCATCACTA | ATCCTTTCACGACCATCT |
| *Casp9* | CTGAGGCAAGCCATAATCG | AGAGGACATGGGAATAGCGT |
| *TNFα* | GCGCTTTTCTGAATCCTACG | TGCCCAGTCTGTCTCCTTCT |
| *IL1β* | GAACAGAATGAAGCACATCAAACC | ACGGCACTGAATCCACCAC |
| *IL6* | TCAACTTCTCCAGCGTGATG | TCTTTCCCTCTTTTCCTCCTG |
| *β-actin* | TCGAGCAGGAGATGGGAACC | CTCGTGGATACCGCAAGATTC |
